# Monkeypox outbreak in the post-eradication era of smallpox

**DOI:** 10.1186/s43162-023-00196-2

**Published:** 2023-02-03

**Authors:** Naga Vishnu Kandra, Anjaly Mary Varghese, Praveen Kumar Uppala, Upendrarao Uttaravelli, Butti Lavanya, S. K. M. Shabana, Venkata Saibaba Somarouthu, Murali Krishna Balijepalli

**Affiliations:** 1 Department of Pharmacology, Santhiram Medical College & General Hospital, Nandyal, Kurnool, Andhra Pradesh India; 2 Employment ID (IPC-291), NCC-PvPI, Indian Pharmacopeia Commission, Ghaziabad, India; 3 Department of Pharmaceutical Analysis, Sri Sivani College of Pharmacy, Srikakulam, Andhra Pradesh India; 4 Department of Hospital & Clinical Pharmacy, K V K College of Pharmacy, Surmaiguda, Hyderabad, India; 5 Department of Pharmaceutical Analysis, K V K College of Pharmacy, Surmaiguda, Hyderabad, India; 6 Centurion University of Technology and Management (CITM), R Sitapur, Odisha India

**Keywords:** Monkeypox, MPOX, Poxviridae, Endemic, Zoonotic, Sporadic, Skin rash, Lymphadenopathy, Lesions, Surveillance

## Abstract

Human monkeypox (MPOX) which recently hit the headlines is a rare, emerging zoonotic disease, only next to smallpox yet never attended adequately to halt the epidemic outbreak threat. MPOX is caused by *Orthopox virus*, which is a double-stranded, linear DNA virus, transmitted from infected animals, commonly rodents to humans. Monkeypox is endemic to the tropical jungles in Central-West Africa; occasional cases reported in other nations could be due to people traveling from endemic regions of MPOX. Transmission may occur via direct contact with human body secretions, cutaneous or mucosal lesions in the mouth or throat or respiratory droplets, and contaminated objects. Typical MPOX symptoms are fever, lymphadenopathy, skin rashes, intense headache, muscle, back pain, etc. Lesions can range from a few to numerous and may be filled with clear or yellowish fluid that later dries up or crusts, eventually falling off. MPOX is often considered as infrequent and self-limiting; nonetheless, the latest sporadic reports call for urgent vigilance, precautionary preparedness, and immediate response. Paucity of the data available about MPOX virus diversity and incomplete information on validated management protocols instigate a sense of impending danger and loom large as a global health emergency. MPOX is a completely preventable infection, and this article will cater to the need for creating general awareness and developing cutting-edge surveillance measures to curtail the spread of the disease. Genomic investigations of new cases of MPOX must be undertaken to check for mutations which can lead to higher human susceptibility. Local health stakeholders and clinicians should emphasize early identification and give out appropriate treatment as per the existing protocol

## Introduction

The name monkeypox (MPOX) stems from the pioneering discovery of the virus in 1958 from the colonies of monkeys used in research in the Copenhagen State Serum Institute in Denmark. In 1970, Congo identified the index MPOX case in an unvaccinated child while combating to eradicate smallpox. The first MPOX outbreak reported outside Africa was in the USA in 2003, following the importation of infected animals. Since the outbreak, 11 other African provinces have reported MPOX in humans spotlighting the Central-West African regions as endemic where the incidence of travel history to endemic places was correlated. Several MPOX cases were recorded in 109 countries including the non-endemic regions by May 2022 and WHO declared MPOX as a moderate global public health emergency of international concern (PHEIC) on 23rd July 2022. Strangely, the incidence of MPOX cases without travel links is high in the 2022 outbreak. 2022 Global MPOX outbreak has reported more than 77,934 lab-confirmed cases and 3666 probable cases to the WHO. In the recent outbreak, 36 MPOX-related deaths have been reported, and one of them is from India. Seventy-five suspected deaths occurred in Africa overall (mostly in Nigeria and Congo). India reported nine MPOX cases till date, among which Kerala state notified five cases and one related death, four from Delhi. The objective of this review is to create awareness about human monkeypox outbreaks and relevant epidemiological information (https://www.who.int/health-topics/monkeypox/#tab=tab_1).

The World Health Organization (WHO) has initiated steps to control and bring down the contagion from further spreading from the international hotspots. An upswing in the confirmed and suspected MPOX cases is observed currently in European and North American nations. The top 10 most affected countries globally are the USA, Brazil, Spain, France, the UK, Germany, Colombia, Peru, Mexico, and Canada. Together, these countries account for 86.4% of the cases reported globally (https://worldhealthorg.shinyapps.io/MPOX_global/).

Two main genetic variants of MPOX were observed with geographic distinction and definite differences clinically and epidemiologically—the Congo Basin (Central African) clade and the West African clade. The Central African variant is a lethal form, having a case fatality rate (CFR) of nearly 11% and demonstrating man-to-man spread whereas the West African genotype documented a CFR of less than 1% with zero human transmission.

**Fig. 1:**
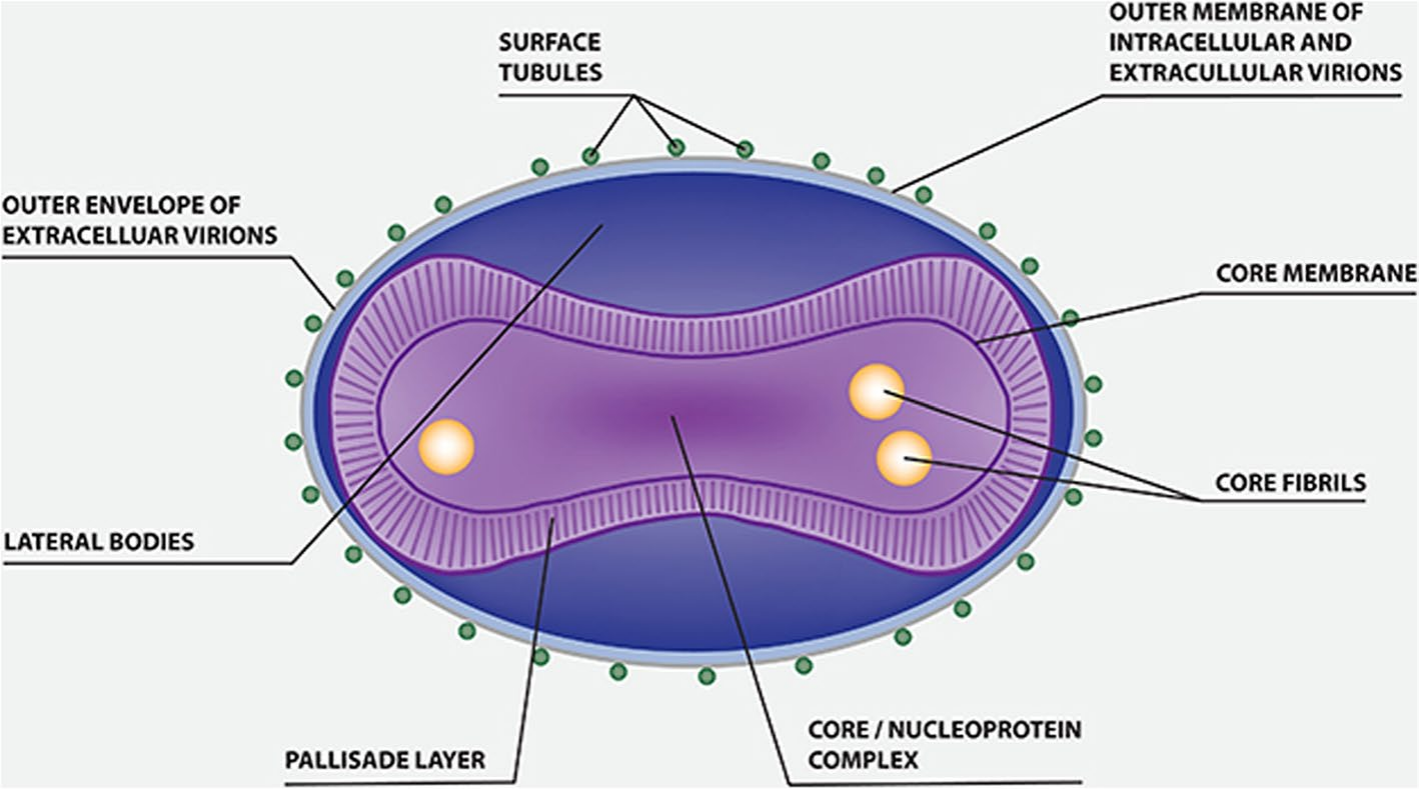
Structure of monkeypox virus

The genus *Orthopoxvirus* belonging to the family *Poxviridae*, includes the chickenpox or varicella virus, smallpox or *Variola* virus (VARV), monkeypox virus (MPOXV), cowpox virus (CPXV), and Vaccinia virus (VACV). MPOX is a close variant of the smallpox virus—*Variola*. The major distinguishing clinical picture of MPOX from smallpox is the prodromal lymph adenopathy after the fever sets in. Smallpox vaccination drive data suggests that this vaccine provided nearly 85% protection against MPOX. Smallpox eradication in the early 1980s led to a suspension of the worldwide smallpox immunization program, making the next-generation vulnerable if MPOX emerges as a silent threat if used as a bioterrorism weapon. Higher risk to healthcare workers persists because of continuous exposure while handling cases suspected or unsuspected [1] (Fig. 1).

Two probable forms of infectious virions are produced by the infected cells—the intracellular mature virus (IMV) and extracellular enveloped virus (EEV). IMV may cause the spread of infection at the cellular level, and EEVs account for the rapid dissemination into the remote body parts of the infected individual.

### Mode of transmission

The suspected natural reservoir for monkeypox disease is the rodents—rats, squirrels, and dormice. Person-to-person transmission may occur through the blood, body fluids, saliva, or respiratory secretions or through exudate or lesion crusts. The recommendations of the Centers for Disease Control and Prevention (CDC) suggest isolation of any suspected case to a separate room and wearing personal protection equipment (PPE) while confronting them. Any personal clothes, bedspreads, cleaning towels, cutleries, dead tissue material, fluids like blood, and pus collected from the lesions must be handled carefully as they are highly infectious. It can be transmitted through sexual contact or vertical transmission from the mother to the fetus. The ongoing outbreak (2022) of MPOX in newly reported countries primarily affects men (homosexual or bisexual). The incubation period of MPOX is the period from the initial viral exposure till symptoms appear, ranging from 7 to 28 days where it harbors in the infected host cell cytoplasm [2].

### Symptomology

The chief symptoms of MPOX infection include acute fever (more than 38.5°C), headache, lymphadenopathy, profound muscle and body pain, backache, and unexplained generalized weakness. After the onset of fever, within 1–4 days, facial lesions erupt before it spreads to the palm and sole. Anogenital lesions are the most typical presentation. Before the lesion resolve completely, it regresses in a peculiar order as follows:

- Macules➔ papules➔vesicles➔pustules➔scabs

There are 2 phases in the symptomatic stage, namely:

- Invasive phase

This includes the initial 1–4 days before the painful or itchy rashes appear. The common symptoms are fever, headache, throat pain, myalgia, backache, lethargy, and lymph node swelling. The lesions may be distributed peripherally, perhaps may cover the whole body depending on the severity and may last up to 4 weeks culminating in the lesion desquamation.

- Eruptive phase

**Fig. 2:**
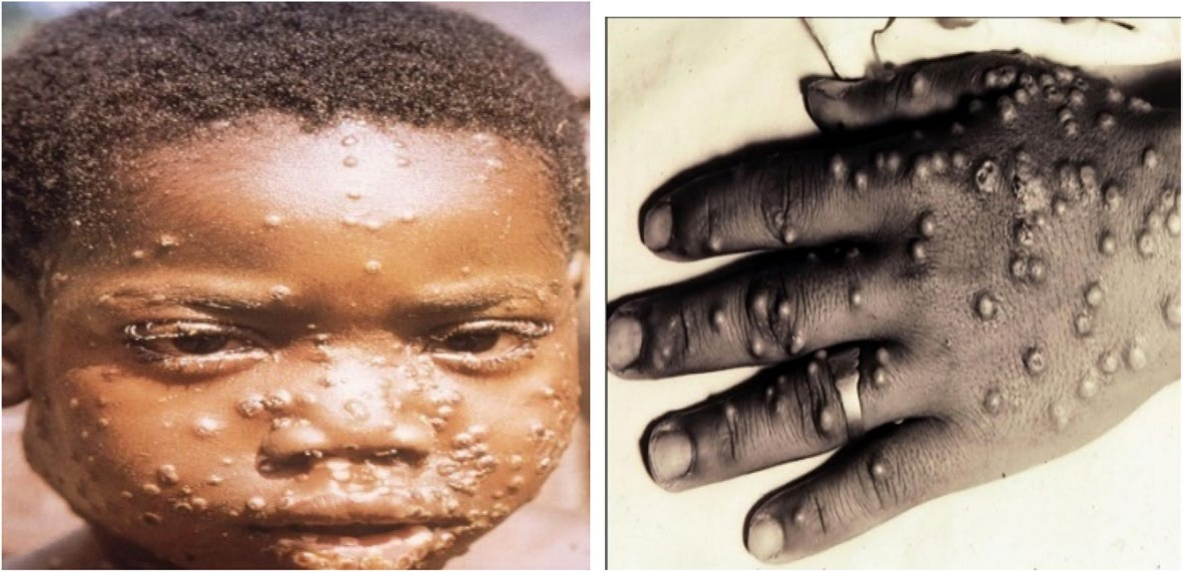
Eruptive phase

**Fig. 3:**
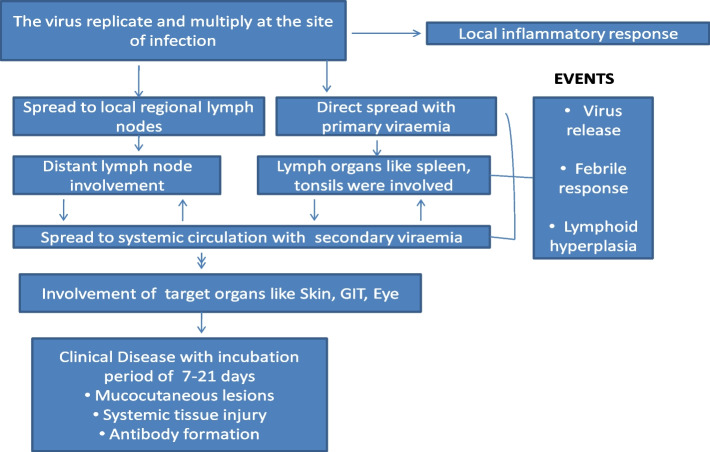
Pathophysiology of monkeypox

In this stage, innumerous cutaneous rashes erupt mainly on the face, hands, feet, perianal area or genitals, and whole body and may affect the mucus membranes. Rashes may appear even on the eyes, mouth, throat, vagina, or anus. It breaks out as tiny lesions that turn into blisters, later may become purulent, and dries up forming scabs. The cutaneous tissue changes are swelling, stiffness, and severe pain. After 3 weeks, these lesions start to disappear. In the first week of developing a rash, the patient is capable of transmitting the infection to others. The sequelae to ocular lesions may result in irreversible loss of vision and scarring of the cornea. Dysphagia may be due to buccal lesions. Rarely, encephalitis is seen in MPOX cases [3] (Figs. 2 and 3).

### Diagnosis

The CDC formulated the case definition criteria for human MPOX during the American outbreak in 2003. MPOX can be clinically diagnosed based on the appearance of the rash. Lymph node enlargement during the early stage differentiates MPOX from chickenpox and smallpox infection. The standard laboratory confirmation can be done by polymerase chain reaction (PCR) method though the results are inconclusive as the MPOX do not survive for long in blood. MPOX cases are classified as suspected, probable, and confirmed. A case is called as “suspected” when the individual has a travel history to any of the endemic regions within the last 3 weeks and shows one or more of the characteristic symptoms (elevated body temperature, body pain, headache, lymph node swelling, and lethargy) with a sudden outbreak of cutaneous rashes. A case is called as “probable” when a person who is clinically suspected of MPOX has an established exposure and temporal association, whereas a confirmed case may be the one confirmed by standard lab diagnostic tests like PCR or genomic sequencing. According to surveillance measures listed in the Indian guidelines, rapid identification of clusters, contact tracing, and testing of all who present with a symptom, infection control must be carried out at the earliest: accordingly, even one single MPOX case must be deemed as an outbreak [4].

### Prophylactic strategies and treatment modalities

Avoid any unprotected physical contact (animal scratch or bite), in case an animal is fallen ill or dead (refrain from consuming such meat, organ meal, or blood) to reduce MPOX transmission. Any animal meat should be consumed only after proper cooking and maintain hand hygiene by using an alcohol-based sanitizer, especially in the endemic regions.

As the incubation period of the monkeypox virus is said to be a maximum of up to 3 weeks, symptoms and temperature monitoring must be done twice per day for 3 weeks. Avoid any skin-to-skin, face-to-face, mouth-to-skin, and mouth-to-mouth close physical contact with a symptomatic person. Avoid any potentially contaminated surfaces or objects used or likely to be used by the infected person. Isolate anyone who develops typical MPOX symptoms. CDC guidelines suggest prophylactic vaccination of the exposed person within 4 days, and disease severity reduction was seen if vaccination was taken well within 2 weeks [5].

CDC recommends vaccination with JYNNEOS (Imvanex or Imvamune), an FDA-approved live attenuated, 3rd generation, non-replicating-Modified Vaccinia Ankara—Bavarian Nordic (MVA-BN) attenuated strain that is incapable of replicating in the human body and elicits a potent immune response, vaccine for the monkeypox and smallpox prevention, and two-shot vaccine (0.5ml each) with at least 4 weeks between doses in people aged 18 years and above. This third-generation vaccine was found to stimulate antibody production and was safer, with superior efficacy when compared with 1st- and 2nd-generation smallpox vaccines. Currently, JYNNEOS (trial mode) is being assessed by the Advisory Committee on Immunization Practices (ACIP) for protective effects in high-risk individuals who have occupational exposure to MPOX or smallpox virus. Tiredness, headache, myalgia, fatigue, and itching were the side effects of JYNNEOS. A history of an allergic reaction after a previous dose of the JYNNEOS vaccine is the contraindication. FDA authorized emergency use of Jynneos to increase vaccine supply to cure MPOX. It is easier to administer. Given during pregnancy or breastfeeding after risk-benefit assessment. Typical serious adverse reactions known for replicating Vaccinia virus strains like myocarditis, encephalitis, and generalized Vaccinia were not observed for the JYNNEOS vaccine.

ACAM2000 is a second-generation smallpox vaccine with the potential use in preventing MPOX during an outbreak. Vaccinia immune globulin intravenous (VIGIV) is approved for the treatment of the Vaccinia virus or Vaccinia vaccination-induced eczema, cutaneous issues, and other abnormal manifestations. The Expanded Access Investigational New Drug (EA-IND) regulations permit VIGIV use in orthopoxvirus outbreaks (including monkeypox) [6]. JYNNEOS is more effective than ACAM2000 because it is contraindicated in immunocompromised, acute eczema, pregnancy cases, and serious adverse effects are seen.

No specific drug is currently available for treatment; only symptomatic therapy and supportive care is the present best option in case management. Generally, MPOX has a low mortality rate (10%) and hence strikes no huge panic among epidemiologists. However, mortality may occur in the 2nd week of MPOX infection in a few cases. Diagnostic difficulties may result in mismanagement and infrequent reporting and also produce inadequate data on the prevalence of MPOX. MPOX is a close variant of the smallpox virus, antiviral medication under the Strategic National Stockpile (SNS) used for treating smallpox infection may be advantageous in MPOX management. Important MPOX drugs are listed below:

- Tecovirimat (TPOXX or ST-246) is an FDA-approved antiviral for smallpox treatment, but not approved for MPOX. Under the EA-IND Protocol, TPOXX can be used on compassionate grounds for the treatment of non-variola orthopoxviruses (including monkeypox) in an outbreak. TPOXX targets and inhibits the major envelope protein VP37 which is essential for the production of extracellular virus. It prevents the virus from leaving an infected cell and thus halts the viral spread in the body.
- Cidofovir (Vistide) is another antiviral approved by the FDA for the treatment of cytomegalovirus (CMV) retinitis in patients with AIDS. CDC holds an EA-IND that allows for the use of Cidofovir for the treatment of orthopoxviruses (including monkeypox) in an outbreak (off-label use). It is often administered intravenously followed by oral probenecid. Cidofovir inhibits viral DNA polymerase and blocks viral DNA synthesis.
- Brincidofovir (Tembexa) is another viral DNA polymerase inhibitor similar to Cidofovir blocking MPOX replication. It was approved by the FDA in June 2021 for smallpox treatment in humans and is presently under EA-IND for managing MPOX infections [7].

## Conclusion

MPOX is a zoonotic viral infection endemic to Central Western Africa and perhaps a large global threat alarming silently. The European Union (EU) biodefense category declares MPOX as a “high threat” or “biosafety level 3.” A sudden multi-country outbreak of MPOX infection has recently evoked suspicion and concern about MPOX’s potential to be misused as a tool for biological warfare. The cry of the hour is to urgently raise awareness among the public and health care providers not to panic but maintain strict vigilance regarding MPOX infection. So far, standard guidelines for clinical management or vaccination available are limited, though human MPOX was reported first in 1970. National and global guidelines for a plan of action must be provided for all reported human MPOX outbreaks to all healthcare workers who are at doubled risk of MPOX exposure. Recently, the guidelines for the diagnosis and management of MPOX were published by the Government of India along with the Ministry of Health and Family Welfare in May 2022 [8].

Taking necessary precautions is the best preventive strategy in any epidemic outbreak. The source of infection must be first identified in each case so as to stop further disease transmission. Containment zones for the identified suspected cases must be created to limit the spread of MPOX which is highly essential to avoid a new surge of a global MPOX pandemic. Immediate focus on accurate information dissemination may help prevent further transmission. Monkeypox is largely a self-limiting disease and low fatality, but high-risk groups can have severe consequences needing necessary medical care. Neonates, children, pregnant women, and immune-compromised people are at a greater risk of serious symptoms and loss of life from MPOX [9]. Strict surveillance measures must be implemented, especially for foreign travelers commuting internationally. Specific MPOX vaccines and targeted therapy against the MPOX are still wanted. Hence, this virus has the potential to pose a serious public health threat worldwide. Efforts must be in the line to strengthen research on MPOX—epidemiology, best practices in prevention, and treatment. Contact tracing of MPOX cases, as well as close contacts of cases must be done according to the national guidelines. Pre- and post-exposure prophylactic immunization is another way to tackle MPOX spread especially in individuals at substantial risk of exposure. The newly approved non-replicating vaccine for MPOX by USFDA was the JYNNEOS vaccine.

## Data Availability

Not applicable to this section.

## Abbreviations

MPOX: Monkeypox
PHEIC: Public health emergency of international concern
EU: European Union
CMV: Cytomegalovirus
SNS: Strategic national stockpile
EA-IND: Expanded access to investigational new drug
VIGIV: Vaccinia immune globulin intravenous
MVA: Modified vaccinia Ankara
PCR: Polymerase chain reaction
CDC: Centers for disease control and prevention
PPE: Personal protection equipment
WHO: World Health Organization
